# Perspectives on plant flavonoid quercetin-based drugs for novel SARS-CoV-2

**DOI:** 10.1186/s43088-021-00107-w

**Published:** 2021-03-24

**Authors:** Manjesh Saakre, Deepu Mathew, V. Ravisankar

**Affiliations:** 1 National Institute for Plant Biotechnology, Indian Agricultural Research Institute, Pusa, New Delhi, 110 012 India; 2 Bioinformatics Centre, Kerala Agricultural University, Thrissur, 680 656 India

**Keywords:** COVID-19, Drug design, Antiviral, Phytomedicine, 3CL^pro^, Zinc, miRNA

## Abstract

**Background:**

The world pandemic COVID-19 caused by SARS-CoV-2 is currently claiming thousands of lives. Flavonoids abundantly present in the fruits and vegetables, especially quercetin, are shown to have antiviral activities.

**Main text:**

This paper reviews the capability of the plant flavonoid quercetin to fight the novel coronavirus and the possibility for drug development based on this. The mode of action explaining the known pathways through which this molecule succeeds in the antiviral activity, action of quercetin on SARS-CoV-2 main protease 3CL^pro^, antiviral activities of its derivatives on human viruses, effect of combination of zinc co-factor along with quercetin in the COVID-19 treatment, and the regulation of miRNA genes involved in the viral pathogenesis are discussed. Proof for this concept is provided following the virtual screening using ten key enzymes of SARS-CoV-2 and assessing their interactions. Active residues in the 3D structures have been predicted using CASTp and were docked against quercetin. Key proteins 3CL^pro^, spike glycoprotein/ human ACE2-BOAT1 complex, RNA-dependent RNA polymerase, main peptidase, spike glycoprotein, RNA replicase, RNA binding protein, papain-like protease, SARS papain-like protease/ deubiquitinase, and complex of main peptidase with an additional Ala at the N-terminus of each protomer, have shown the binding energies ranging between − 6.71 and − 3.37 kcal/ Mol, showing that quercetin is a potential drug candidate inhibiting multiple SARS-CoV-2 enzymes.

**Conclusion:**

The antiviral properties of flavonoid and the molecular mechanisms involved are reviewed. Further, proof for this concept is given by docking of key proteins from SARS-CoV-2 with quercetin.

**Graphical abstract:**

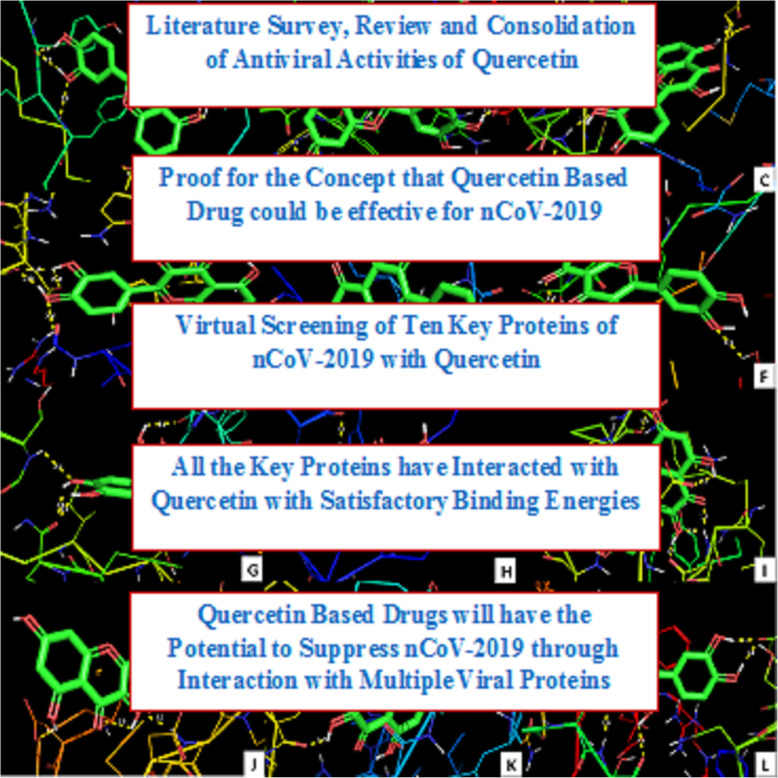

## Background

Coronavirus disease (COVID-19) is a highly contagious disease caused by novel severe acute respiratory syndrome-related Coronavirus (SARS-CoV-2) which has not been reported earlier in humans. During December 2019, this disease was first reported from Wuhan, China. Considering the widespread across the globe, crossing international borders and affecting a large percentage of the population, the World Health Organization has declared COVID-19 as a pandemic. The development of vaccines by institutions such as AstraZeneca/University of Oxford (UK), Institut Pasteur/Merck/Themis (France/USA/Austria), University of Hong Kong (China), CureVac (Germany), Moderna (USA), Inovio (USA), Clover Biopharmaceuticals (China), Novavax (USA), University of Queensland/CSL (Australia), and Sechenov University (Russia, Sputnik V), is in different phases and few are being administered, but no specific treatment protocol has so far been developed. It is indeed the need of the hour to develop rapid diagnostic kits, vaccines, novel therapeutics, and drug-repurposing for COVID-19. Questions on viral transmission, mode of action, immunity, and other critical points need to be answered urgently.

Coronaviruses (CoVs) are comparatively large enveloped viruses with single-stranded positive-sense RNA genome (29,903 bp) [1] packed within a membrane. The membrane is covered with glycoprotein spikes, offering crown-like appearance to the coronaviruses [2]. CoVs are the largest group of viruses which are the members of Nidovirales order that includes Coronaviridae, Arteriviridae, and Roniviridae families. The Coronaviridae is subdivided into alpha, beta, gamma, and delta groups. Its genome possesses a 5′ cap and a 3′ poly-A tail, permitting it to act as an mRNA for translation of the replicase polyproteins. The nonstructural proteins (Nsps) are encoded by a replicase gene that comprises ~ 20 kb, and the structural and accessory proteins are coded by about 10 kb of the viral genome [3].

To treat COVID-19, researchers are aggressively struggling to develop the vaccines and efficient drugs, which are all still in clinical trials and may take some time to reach public. Initially, hydroxychloroquine and azithromycin have been repurposed to reduce viral load in COVID-19 patients [4], but these drugs were shown less effective subsequently. In addition, molecular docking studies are also undergoing to predict suitable drugs to inhibit the virus. Sequence analysis, molecular docking, and *in vitro* studies have suggested that antiviral drugs such as Sofosbuvir, Ribavirin (used to treat hepatitis C), and Remdisivir (a nucleotide analog, for treating Ebola virus disease and Marburg virus infections) can be used against the new strain of coronavirus and came up with promising data [5]. Anti-parasite drug ivermectin was also reported to inhibit the replication of SARS-CoV-2 *in vitro* [6]. Even with all these independent reports, it has not been easy to ascertain if the outcome is complete viral inhibition or minimal benefit at heavier adverse effects. For instance, hydroxychloroquine is widely used to treat malaria, lupus erythematosus and rheumatoid arthritis. But this drug has adverse effects such as nausea, heart problems, headache, and occasional stomach cramps with mild diarrhea.

Competency of many plant extracts to counteract various viral infections has elevated the expectations about them being the future antiviral agents. Antimicrobial effects of plant extracts are mainly due to the secondary metabolites such as tannins, alkaloids, and flavonoids. Among the secondary metabolites, flavonoids are found to have the maximum antiviral activity [7, 8]. Flavonoids are characterized as the largest group of natural polyphenols and the most widespread class of compounds in vegetables, fruits, and plant-based beverages. At least 8000 compounds with flavonoid structure have been known, among which many are responsible for the beautiful colors of flowers, fruits, and leaves [9].

Flavonoids positively impact the variables associated with atherosclerosis, including vascular reactivity, increase in platelet count and lipoprotein oxidation [10]. Antioxidant, antithrombotic, anti-inflammatory, and hypolipidemic conditions developed with the consumption of flavonoid-rich diet, lowers the cardiovascular mortality [11]. Flavonoids also have antibacterial and anti-inflammatory effects. Thus, recently, therapeutic and nutritional research focus a lot on the flavonoids. Quercetin is one of the essential natural flavonoids present in most of the fruits, vegetables, and in few plant leaves. It prevents cardiovascular diseases and is used for treating the neurodegenerative diseases such as Alzheimer’s and Parkinson’s diseases [12]. This molecule additionally offers protection against cancer, apoptosis, arthritis, ulcer, gastritis, diabetes, bladder infections and possesses antibacterial and anti-viral activities [12–19]. The broad spectrum antiviral activity of quercetin has been well demonstrated by various scientific investigations and found as a very effective compound against many human viral diseases. In this review we discuss the mode of action and antiviral properties of quercetin. We also focus on predicting its biological actions including the inhibition of key enzymes of SARS-CoV-2 and speculated the possibility of a combinatorial drug approach by combining quercetin with zinc ions.

## Prospects of quercetin as drug for nCoV

### Quercetin and its modes of action

Quercetin is abundantly found in fruits such as apple and citrus and in vegetables such as onion, broccoli, kale, and tomato [20, 21]. Also, papaya leaves, green and black tea leaves, buckwheat, seeds, and grains contain considerable amounts of quercetin. Quercetin comprises two benzene rings (A and B) attached by a distinctive carbon ring C (C6–C3–C6) with a benzopyrone skeletal structure [22]. This plant flavanol is an antioxidant with multiple OH groups around the periphery, and it neutralizes the potentially damaging free radicals through the donation of hydrogen atoms. This way, quercetin justifies its antioxidant property. Quercetin acts as a strong reducing agent and protects body tissue against oxidative stress, and its antioxidant activity increases cell survival rate [23]. Its prooxidant activity promotes apoptosis in cancerous cells whereby preventing the tumor proliferation. The tumor growth inhibition assays have reported that proliferation of K562 human chronic myelogenous leukemia, HT-29 human colon adenocarcinoma, and human breast adenocarcinoma cell lines have been inhibited by quercetin [24]. Quercetin is a potential scavenger of reactive oxygen species and a strong antioxidant which chelates metal ions [25].

Another possible mode of action proposed is its interaction with various enzymes by targeting the key amino acid residues in the active sites. The anti-inflammatory activity of quercetin is attributed to the inhibition of cyclooxygenase enzyme that is responsible for the synthesis of prostanoids, including thromboxane and prostaglandins [26]. In addition, quercetin along with quercetin monoglucosides reduces the development of atherosclerosis, by inhibiting the enzyme 15-lipoxygenase which modifies low-density lipoprotein through oxidation [25]. Homology modeling, molecular docking and *in vivo* studies have shown that the antiviral properties of quercetin are through the inhibition of key enzymes of viral replication and packaging, reducing the viral load in the host cells. Mice receiving quercetin oral treatment were found protected against Mengo_M_, Col. SK, MM, intraperitoneal encephalomyocarditis, and Mengo_M,L_ viral infections. This study had shown that the action of macrophages is necessary for quercetin to be effective rather than thymus [27].

### Antiviral activities of quercetin and its derivatives

Quercetin interferes with the viral replication at several stages. Through inhibition of PI-3 kinase, quercetin blocks endocytosis in viruses. The transcription of viral genome is stopped by the inhibition of RNA-dependent RNA polymerase, and by promoting the cleavage of eIF4G, translation of viral proteins is disrupted. In addition, through enhanced mitochondria-associated antiviral responses, quercetin is found to increase the viral clearance [28]. All these reduce the pro-inflammatory responses.

In rauscher murine leukemia virus (RLV) and human immunodeficiency virus (HIV), it was shown that the quercetin was competent to inhibit the viral reverse transcriptases [29]. Suppressive action of tumor necrosis factor (TNF) against encephalomyocarditis virus (EMCV) and vesicular stomatitis virus (VSV) was significantly enhanced by quercetin. TNF induces the expression of 2',5'-oligo-adenylatesynthetase (RNase L system is an innate immunity pathway responding to a pathogen-associated molecular pattern) in combination with quercetin [30].

Quercetin also inhibits common cold-causing Rhinoviruses (RV) by reducing the replication of negative and positive strand RNA, and translation of capsid proteins [28]. Furthermore, quercetin inhibits the infection by various strains of influenza viruses, by interacting with the HA2 subunit of glycoprotein hemagglutinin of the viruses which plays an important role in the early stages of infection [31]. This flavonoid has significantly lowered the herpes simplex virus 1 (HSV-1) infection in cell lines (Raw 264.7) by inhibiting the expression of HSV proteins such as glycoprotein D and infected cell protein (ICP0) and genes such as ICP0, UL13, and UL52 [32]. Similarly, cell line studies have proven the antiviral properties of quercetin against equid herpesvirus 1 [33] and Japanese encephalitis virus [34].

Often quercetin and its derivatives are found to complement each other in their antiviral activities. Quercetin in combination with quercitrin exhibits good anti-Dengue virus type-2 activity and reduced cytotoxicity [35]. Substituted forms of quercetin are found to have enhanced antiviral activity [36]. Quercetin and its derivative quercetin 3-O-glycosides (Q3G) show good anti-Mayaro virus [37] and anti-influenza A virus activities [38]. In addition, this glucoside derivative has antiviral efficiency against Ebola virus, both *in vitro* and *in vivo*. Q3G affects viral entry more specifically at glycoprotein-mediated step in the viral life cycle [39]. The ELISA results revealed that Q3G inhibits the replication of Zikavirus in a dose-dependent manner, as evidenced by the reduction in the expression of nonstructural protein 1 [40].

Quercetin 7-rhamnoside (Q7R) and other structural analogues, quercetin, apigenin, luteolin and catechin, were demonstrated to have suppressive activity against porcine epidemic diarrhea virus [41]. The antiviral property was through their interference in the early stage replication of the virus. Similarly, quercetin 3-rhamnoside was also found to suppress the influenza A virus replication [42]. The target proteins of quercetin in common human viruses, leading to its antiviral properties, are summarized in Table 1.

**Table 1:** Target proteins for quercetin in human viruses and their functions

Sl. no.	Virus	Protein targets for quercetin	Function of target protein	Reference
1.	Rhinovirus	Capsid protein	Mainly protects the virus nucleic acid from digestion by nucleases	[28]
2.	Vesicular stomatitis virus (VSV) and encephalomyocarditis virus (EMCV)	2', 5'-oligo-adenylate synthetase^a^	RNase L system is an innate immunity pathway that responds to a pathogen-associated molecular pattern	[30]
3.	Influenza A virus	HA2 subunit of hemagglutinin	HA2 subunit is a part of hemagglutinin responsible for viral-host cell fusion	[31]
4.	Herpes simplex virus 1	gD and ICP0	gD is a structural component of the HSV envelope which is essential for virus entry into host cells. ICP0 is crucial for virus to progress lytic infection and for reactivation from latency	[32]
5.	Ebola virus^b^	-	-	[39]
6.	Zikavirus^b^	Nonstructural protein 1	NS1 is known to be a major host-interaction molecule involved in virus replication, pathogenesis, and immune evasion	[40]
7.	SARS-CoV	3CLpro	Coronavirus main protease plays key virus replication and packaging	[43]
8.	Enterovirus 71	EV71 protease	Play an important role in virus replication and packaging	[44]
9.	Dengue 2 virus	Viral non-structural 2B and 3 (NS2B-NS3) protease complex	Essential for the polyprotein synthesis precursor prior to the assembly of the viral complex	[45]

^a^Protein over expressed; ^b^ Quercetin 3-βO-D-glucoside was used in the study

### Quercetin targets SARS-CoV-2 main protease 3CL^pro^

The 3C-like protease (3CL^pro^) is a cysteine protein known as main protease in coronaviruses. This enzyme is essential for the cleavage and processing of viral polyproteins into effector proteins, facilitating virus replication and packaging within the host cells [2]. The genomes of SARS-CoV and SARS-CoV-2 have only moderate sequence similarity (79.0%) yet *3CL*^*pro*^ gene is >95.0% similar and hence the inhibitors of SARS-CoV 3CL^pro^ would be promising drugs for nCoV. Quercetin had >80.0% *in vitro* inhibition activity, with IC_50_ value of 73 μM, on the recombinant 3CL^pro^ protein expressed in *Pichia pastoris* [43].

Molecular docking, SPR/FRET (Surface Plasmon Resonance/ Fluorescence Resonance Energy Transfer) based bioassays, and mutagenesis studies have revealed that the quercetin derivative Q3G acts as a potential inhibitor of the 3CL^pro^ of SARS-CoV [46]. Molecular modeling and mutation studies have shown that Gln_189_ is one of the determining amino acid residues in catalytic pocket of SARS-CoV 3CL^pro^ where Q3G mainly interacts, leading to enzyme inhibition.

Recent molecular docking studies and *in silico* screening of herbal medicines had also suggested that quercetin is one of the potential inhibitors for 3CL^pro^ of SARS-CoV-2 [47–49]. A traditional Chinese medicine study reported that quercetin from *Toona sinensis* extract has a potent anti-SARS-CoV property which inhibits the viral cellular entry, adsorption, and penetration into the target cells [50]. Quercetin also potentially inhibits the activity of enterovirus EV71 protease 3CL^pro^, decelerating the viral replication. Molecular modeling and docking studies have predicted that quercetin gets inserted into the substrate-binding pocket of EV71 3CL^pro^, blocking substrate recognition, protein activity, and thus the virus replication [44]. These investigations point that the quercetin effective for suppressing SARS-CoV 3CL^pro^ can target SARS-CoV-2 3CL^pro^ also. Currently, other SARS viral proteins such as PL^pro^, nonstructural proteins (NSPs), and RNA-directed RNA polymerase are targeted in molecular docking with quercetin and in *in vitro* studies. Quercetin is reported to potentially dock with the NS2B-NS3 protease complex of dengue 2 virus (DENV-2) [45].

The US Food and Drug Administration (FDA) had approved quercetin as safe for human consumption, as its cytotoxicity is very low (the national drug code numbers 65448-3085 and 65448-3005 [51]. The research and clinical trials are in progress to establish quercetin as a potential drug against SARS-CoV-2. So far, there are two registered clinical trials of quercetin for COVID-19, registered at http://www.clinicaltrials.gov. The study “The possible effect of quercetin on prophylaxis and treatment of COVID-19” (ClinicalTrials.gov Identifier: NCT04377789), started on 20 March, 2020 with 50 enrolled participants and considers the antioxidant and antiviral properties of quercetin and the possibility of developing drugs with no adverse effects.

### Combination of quercetin with zinc ions

Even though zinc is an essential cofactor for many cellular enzymes, it is found to block the RNA-dependent RNA polymerase (RdRp) of several viruses [52, 53]. Higher levels of intracellular zinc increases the intracellular pH, affecting the RdRp activity and decreasing the viral replication. *In vitro* studies have shown that Zn^2+^ ions can inhibit SARS-CoV RdRp (nsp12) at template binding and at elongation step. Further, zinc ions can block the initiation step of RNA synthesis of equine arteritis virus (EAV) [52]. Moreover, the same study has reported that the combination of zinc ions and the zinc ionophore pyrithione efficiently inhibited coronavirus and arterivirus replication in cell cultures. In another investigation, both *in vitro* and *in vivo* experiments had shown that zinc ions can block hepatitis E virus replication by inhibiting the activity of viral RdRp [53]. The charged zinc ions require a transporter for the influx into the cell, and quercetin acts as an ionophore that mediates zinc influx into the cells through plasma membrane [54]. While correlating these results, it is evident that the combination of quercetin and zinc is a potential strategy against SARS-CoV-2, imparting greater antiviral efficacy at a lower cytotoxicity. Quercetin possibly plays a dual role by directly inhibiting the viral 3CL^pro^ and indirectly supporting the blocking of RdRp by acting as ionophore for zinc influx.

Considering the lower side effects, many researchers have attempted the possibility of developing nCoV drugs from natural products [55–57]. A second study on zinc and quercetin complex in COVID-19 treatment, registered at http://www.clinicaltrials.gov, “The study of quadruple therapy zinc, quercetin, bromelain, and vitamin C on the clinical outcomes of patients infected with COVID-19” (Clinical Trials.gov Identifier: NCT04468139), started on 20 June, 2020, uses a quadruple therapy of zinc, quercetin, bromelain, and vitamin C on COVID-19 patients. The formulation works on the interplay between quercetin and zinc to block the SARS-CoV-2 RdRp with the assistance from the anti-inflammatory agent bromelain and antioxidant and immune booster vitamin C.

### Quercetin regulates microRNA genes

The small noncoding RNAs named miRNAs perform critical roles in developmental biology and are shown to silence the viral replication in a sequence-specific manner. Few studies correlating quercetin and miRNAs have placed miRNAs directly in the defensive effect. The available data suggests that quercetin regulates the expression of miRNAs which are responding to specific diseases particularly for cancers. For instance, differential expression of multiple miRNAs was observed in lung tumor tissues when feeding with quercetin-rich diet was done. Interestingly, more frequent consumption of quercetin-rich foods was strongly associated with the expression of miRNAs belonging to the let-7 miRNA family [58]. Quercetin treatment has resulted in the upregulation of miRNA let-7c which inhibits pancreatic cancer progression by post-transcriptional activation of Numb-like (NumbL) gene [59]. In another investigation, quercetin and quercetin-3-O-galactoside, when applied in equal proportion, has downregulated the oncogenic miRNA-27a in 786-O renal cancer cells [60]. These studies showed that quercetin can also induce the expression of miRNA genes, especially in cancer cells.

**Fig. 1:**
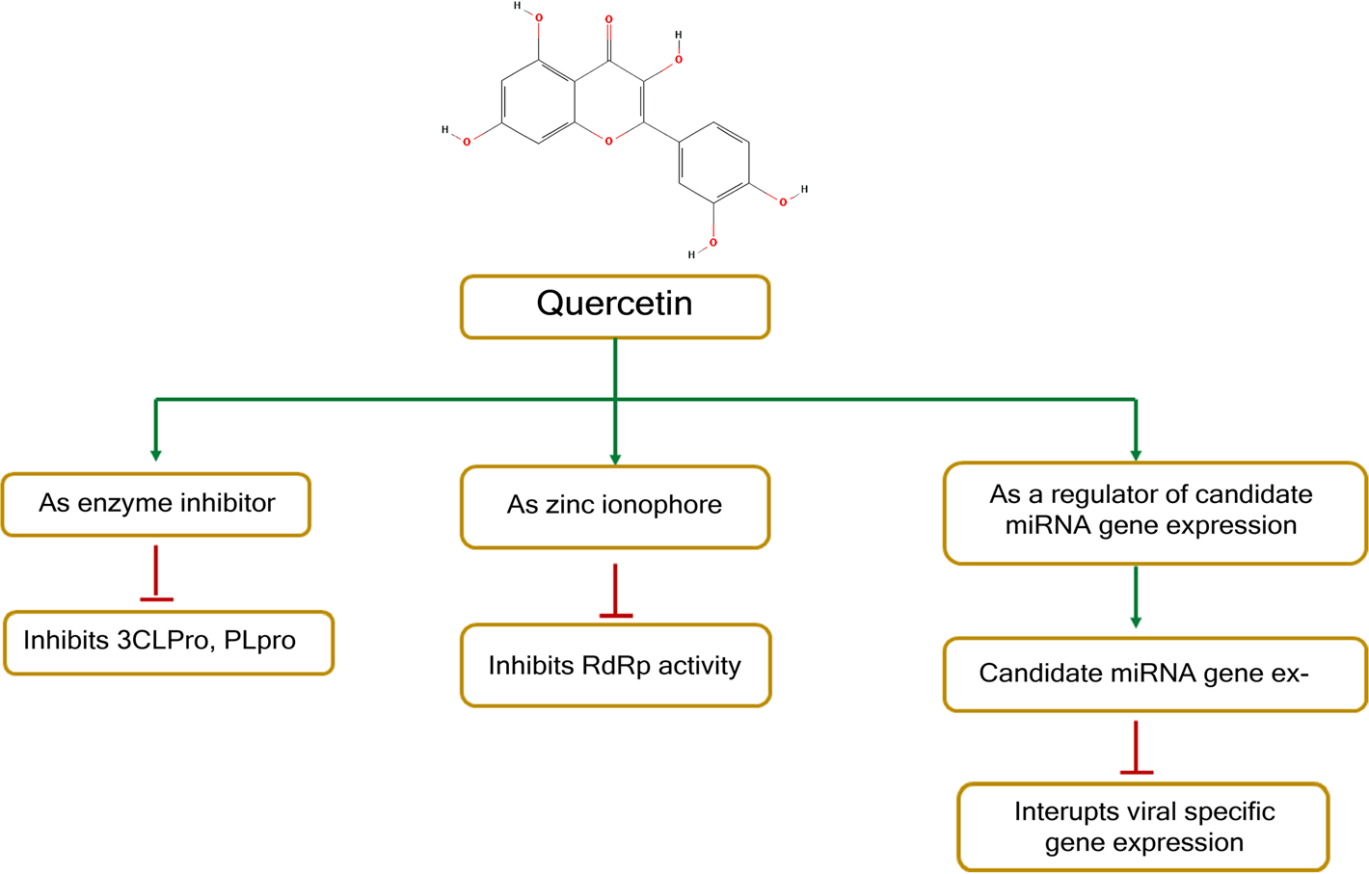
Multiple paths through which quercetin effects its antiviral properties

In recent times, miRNA-based therapies have gripped the attention of researchers and a few precise *in silico* investigations are being initiated to link the genome of SARS-CoV2 with human miRNome, to understand the role of miRNAs in SARS-CoV-2 infections. An investigation based on computational studies has speculated that miR-27b plays an important regulatory role in SARS-CoV-2 infection, and it has a strong correlation with ACE2. Among the miRNAs analyzed, miR-27b was unique with a target gene in the Indian SARS-CoV2 genome which was missing in the strains from other countries [61]. Computational analyses have predicted that the genes *Envelope*, *ORF6*, and *ORF1ab* are targeted by different human mature miRNAs [62]. An *in silico* hybridization-based analysis has predicted that 22 potential miRNAs from five genomes of SARS-CoV2 are connected with 12 human miRNAs. Hsa-mir-1267, hsa-mir-1-3p, and hsa-mir-5683 are human candidate miRNAs reported to share between all the five viral SARS-CoV2 miRNAs [63]. Regrettably, there are lacking evidences suggesting that quercetin can regulate the expression of candidate miRNAs that are involved in neutralizing the viral infection. In this session, we are hypothesizing and correlating that quercetin might be regulating human candidate miRNAs which are possibly target SARS-CoV-2 genomes. The possible multiple paths through which quercetin effects its antiviral properties are presented in Fig. 1. Detailed *in vitro* and *in vivo* studies are necessary to validate the candidate miRNAs involved in SARS-CoV-2 gene expression and to demonstrate the participation of quercetin in their regulating.

## Proof of concept

### Methods

In 2020, we accessed the Google Scholar, Web of Science, and PubMed to identify publications with the search string: “quercetin*”, “flavonoid*”, “COVID-19*”, “key proteins*”, “nCoV*”, “phytomedicine*”, “drug design*”, “antiviral*”, “phytocompound*”, “virtual screening*”, “miRNA*”, “zinc*”, etc.

To give the proof for our concept that quercetin could be a potential drug against novel coronavirus, we have followed the molecular docking methodology. Three-dimensional structures of ten key proteins of coronavirus were retrieved from PDB. 2019-nCoV main protease (PDB IDs: 6LU7, 6Y84, 6YB7), 2019-nCoV receptor-binding domain of surface spike glycoprotein (S protein, PDB ID: 6M17), 2019-nCoV spike glycoprotein (PDB ID: 6VXX), 2019-nCoV RNA replicase enzyme (PDB ID: 6W9Q), 2019-nCoV RNA-binding protein (PDB ID: 6W4B), 2019-nCoV papain-like protease (PDB ID: 6W9C), SARS coronavirus papain-like protease/ deubiquitinase (PDB ID: 3E9S), 2019-nCoV RNA-dependent RNA polymerase (PDB IDs: 7BTF, 6M71, 6NUR), SARS coronavirus main peptidase (PDB ID: 2A5I), and SARS coronavirus main peptidase with an additional Ala at the N-terminus of each protomer (PDB ID: 2GTB) were the proteins used in the study. The chains of each protein/ protein complex were identified as described in PDB. The active sites of the proteins were identified using CASTp3.0 webpage. The 3D structures of the proteins and the ligand were loaded into molecular docking software AutoDock 4.2. The active sites identified were provided in AutoDock, and the docking grid was set accordingly and the docking was performed.

### Results

**Fig. 2:**
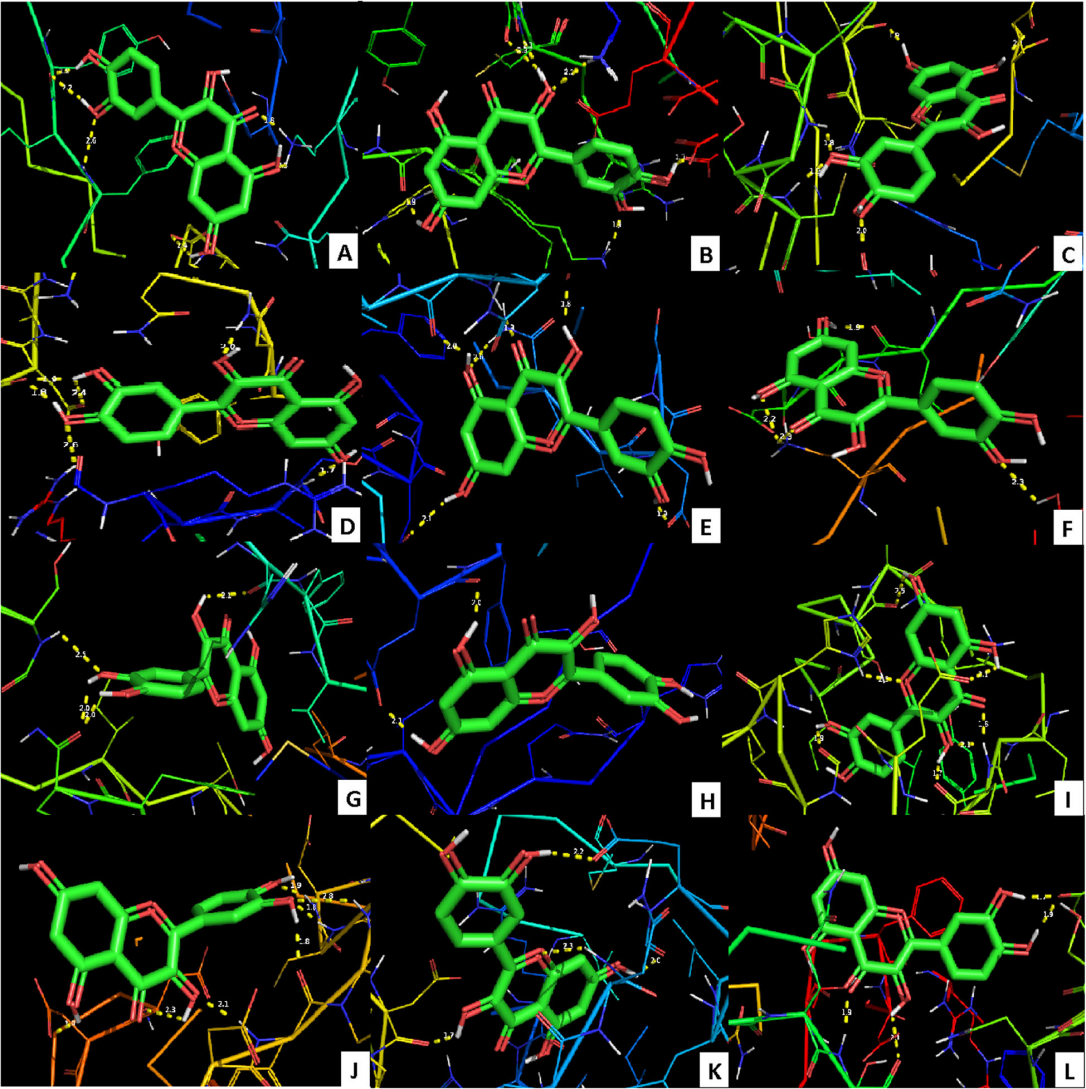
Molecular docking of key proteins with quercetin. **a** 2019-nCoV main protease (PDB ID: 6LU7). **b** 2019-nCoV main protease (PDB ID: 6Y84). **c** 2019-nCoV main protease (PDB ID: 6YB7). **d** 2019-nCoV receptor-binding domain of the surface spike glycoprotein (PDB ID: 6M17). **e** 2019-nCoV spike glycoprotein (PDB ID: 6VXX). **f** 2019-nCoV RNA replicase enzyme (PDB ID: 6W9Q). **g** 2019-nCoV RNA-binding protein (PDB ID: 6W4B). **h** 2019-nCoV papain-like protease (PDB ID: 6W9C). **i** SARS coronavirus papain-like protease/ deubiquitinase (PDB ID: 3E9S). **j** 2019-nCoV RNA-dependent RNA polymerase (PDB ID: 7BTF). **k** SARS coronavirus main peptidase (PDB ID: 2A5I). **l** SARS coronavirus main peptidase with an additional Ala at the N-terminus of each protomer (PDB ID: 2GTB)

The binding energies of few key target protein conformations with the quercetin were recorded (Table 2). The main protease or 3CL^Pro^ of novel coronavirus is a key enzyme that mediates the viral replication mediates the viral replication and transcription [64] and has been identified as a potential drug target by many researchers [72]. Three confirmations available at PDB have been independently docked with quercetin and found that all the confirmations dock with the ligand. The residues interacted were LYS88, TYR101, LYS137, GLY138, ASP289, GLY143, and CYS145 with the docking energy − 6.71 kcal/Mol (Fig. 2a–c).

**Table 2:** Molecular docking of various proteins of coronavirus with quercetin ligand

Sl. no.	Protein	PDB ID and chain	Best binding energy (kcal/Mol)	Interacting residues having H bond	Protein structure at PDB	Remarks
1	2019-nCoV main protease (3CL^pro^)	6LU7.A, 6Y84.A, 6YB7.A	− 6.71	LYS88, TYR101, LYS137, GLY138, ASP289, GLY143, CYS145		Non-structural polyprotein 1ab. Key enzyme mediating viral replication and transcription [64]. The active site residues from individual PDB structures were separately predicted using CASTp, each structure was docked separately, and interacting residues were identified.
2	2019-nCoV RBD of spike glycoprotein/ human ACE2-B^O^AT1 complex	6M17.E	− 5.56	SER349, LEU441, ASN450		Angiotensin-converting enzyme 2 (ACE2) is the human cellular receptor for SARS coronavirus (SARS-CoV) by interaction with the receptor-binding domain (RBD) of the surface Spike glycoprotein (S protein) of SARS-CoV-2 [65]
3	2019-nCoV spike glycoprotein	6VXX.A	− 5.19	ASP88, ASP198, ILE233, ILE235		Homotrimer protein [66]
4	2019-nCoV RNA replicase	6W9Q.A	− 5.89	LEU45, THR109		Non-structural protein 9
5	2019-nCoV RNA binding protein	6W4B.A	− 5.44	PRO58, THR68		Non-structural protein 9
6	2019-nCoV papain-like protease	6W9C.A	− 4.18	GLN30		Hydrolase enzyme, Homo 3-mer - A3
7	SARS coronavirus papain-like protease/ deubiquitinase	3E9S.A	− 3.37	LYS158, ASP165, THR169		Replication of SARS coronavirus requires proteolytic processing of the replicase polyprotein by two cysteine proteases, a chymotrypsin-like protease (3CL^pro^) and a papain-like protease (PL^pro^) [67, 68]
8	2019-nCoV RNA-dependent RNA polymerase	7BTF.A, 6M71.A, 6NUR.A	− 5.41	TYR619, CYS622, ASP623, ASP761, SER841		Non-structural protein 12, catalyzes the synthesis of viral RNA and thus plays a central role in the replication and transcription cycle of COVID-19 virus [69]. The active site residues from individual PDB structures were separately predicted using CASTp; each structure was docked separately and interacting residues were identified.
9	SARS coronavirus main peptidase	2A5I.A	− 5.12	TYR54, GLU55, ASP187		Hydrolase enzyme [70, 71]
10	SARS coronavirus main peptidase with an additional Ala at the N-terminus of each protomer	2GTB.A	− 5.20	THR111, ASP153

Angiotensin-converting enzyme 2 (ACE2)-B°AT1 complex on human cell surface recognizes SARS-CoV by interaction with the receptor-binding domain (RBD) of the surface spike glycoprotein [65], and hence RBD is also a potential drug target [73]. Docking RBD with quercetin had shown good interaction at SER349, LEU441, and ASN450 with the binding energy of − 5.56 kcal/Mol (Fig. 2d). Similarly, spike glycoprotein of novel coronavirus is a promising drug target [74], and this protein was also docked with the quercetin. Residues ASP88, ASP198, ILE233, and ILE235 were found to interact with the quercetin with a good binding energy of − 5.19 kcal/Mol (Fig. 2e). ACE2 is a transmembrane protein localized at the lung alveolar epithelial cells. It acts as a monocarboxypeptidase that cleaves a single C-terminal residue from angiotensin II, producing angiotensin [75]. ACE2 was identified as a receptor for spike proteins (S protein) of SARS-CoV-2 to penetrate into host cells. It is predicted that SARS-CoV-2 infection can be introverted by inhibiting the interaction between S protein and the host ACE2. Therefore, quercetin may prevent the entry of SARS-CoV-2 into cells by impairing the binding of viral S protein to ACE2 receptor. An *in vitro* study showed that quercetin and its derivatives inhibit 42–48% recombinant human ACE2 activity. Their results inferred that quercetin with an IC_50_ of 4.48 μM found as the most potent inhibitor of recombinant human ACE2 among the other polyphenols tested [76]. Our docking studies assist *in vitro* and *in vivo* studies by predicting the target residues that bind quercetin.

According to Liu et al. [76], inhibition of ACE2 may be undesirable because functional ACE2 inhibits inflammation by reducing activation of the angiotensin II type 1 receptor pathway. SARS-CoV-2 uses ACE2 as a receptor to enter cells, and the resulting proteolysis of ACE2 contributes to lung damage. Disrupting S protein and ACE2 interactions might prevent SARS-CoV-2 entry to cells but inhibiting ACE2 activity could be detrimental to infection recovery. Molecular docking analysis also showed that quercetin has high binding affinity to the viral spike protein. Quercetin is listed as one of the top-scoring ligands for S protein-ACE2 receptor interface, and this has undergone regulatory review in the USA [77].

Key enzyme of novel coronavirus RNA replicase was also docked with quercetin. The protein interacted with the quercetin at LEU45 and THR109 residues with a binding energy of − 3.89 kcal/Mol (Fig. 2f). Replicase enzyme machinery in coronoviruses is a heavy protein complex, and any modification to this complex could deactivate the virus multiplication and transcription [78].

In RNA viruses, RNA-binding proteins play an important role in the control of many cell processes from replication to translation. The docking of RNA-binding protein 6W4B from novel coronavirus with quercetin has shown the active interaction at PRO58 and THR68 residues with a good binding energy of − 5.44 kcal/Mol (Fig. 2g).

In coronaviruses, replicase polyprotein complex has to be initially proteolytically processed to gain its functionality. This is done by two cysteine proteases, a chymotrypsin-like protease or main protease (3CL^pro^) and a papain-like protease (PL^pro^) [67, 68]. Additionally, PL^pro^ inhibits the type I interferon signalling pathway through interaction with the STING-TRAF3-TBK1 complex leading to the reduced immunity [79]. Thus, this protein complex can be a potential drug target. Docking of 2019-nCoV papain-like protease with quercetin has shown good interaction at GLN30 with a binding energy of − 2.18 kcal/Mol (Fig. 2h). In AutoDock, the binding energies shall be lower when the docking is performed with smaller ligands such as quercetin. Similarly, docking with the SARS coronavirus papain-like protease/ deubiquitinase enzyme was also attempted. This interaction also had shown good interaction at LYS158, ASP165, and THR169 residues with a binding energy of − 3.37 kcal/Mol (Fig. 2i).

RNA-dependent RNA polymerase (RdRp) is a very important protein complex for replication of coronavirus in host cell, and it is accepted as a potential drug target [80]. Chemical structures from traditional Chinese medicinal compounds which had good docking scores with RdRp of SARS CoV-19 were found to have good antiviral activity [81]. Xu et al. [82] reported that ASP760 and ASP761 are the key amino acid residues constituting the RdRp catalytic domain. In this study, three confirmations of this enzyme have separately docked with quercetin and TYR619, CYS622, ASP623, ASP761, and SER841 were found to offer good interaction with a binding energy of − 5.41 kcal/Mol (Fig. 2j). Thus, to change the confirmation of target viral proteins and to inhibit them, binding of quercetin can be at any active site of the virus protein and not necessarily at the sites which interact with the human proteins. These results show that quercetin is a strong inhibitor of viral RdRp, and further *in vitro* and *in vivo* studies can prove the potential of this wonder molecule. Targeting the RdRp active site ASP761 by quercetin could be a potential therapeutic choice for inhibition of SARS-CoV-2 replication. Aftab et al. [83] also used computational approach and predicted that ASP761 also strongly bind antiviral drug Galidesivir (a nucleotide analog, for treating Ebola virus disease, Marburg virus disease, and Zika virus) which shows that quercetin honor a similar potential as Galidesivir. Galidesivir is also under phase 1 human trial in Brazil for COVID-19 (ClinicalTrials.gov Identifier: NCT03891420).

The SARS coronavirus main peptidase plays an essential role in the life cycle of the virus [70] and is a primary target for the development of anti-SARS agents [71]. SARS coronavirus main peptidase interacted with quercetin at TYR54, GLU55, and ASP187 residues with a binding energy of − 5.12 kcal/Mol (Fig. 2k). Confirmation of the SARS coronavirus main peptidase with an additional Ala at the N-terminus of each protomer was additionally docked with quercetin, and this also has shown interaction at THR111 and ASP153 residues with a binding energy of − 5.20 kcal/Mol (Fig. 2l).

Thus, it is shown that quercetin is a potential drug molecule for the treatment of COVID-19 in humans. All the ten key proteins we have experimented have shown good interaction at multiple amino acid residues with satisfactory binding energies. Further steps in drug formulation starting from *in vitro* and *in vivo* animal model experiments have to be initiated urgently.

## Conclusion

COVID-19 is currently the world’s worst pandemic, instantly demanding the therapeutic molecules and vaccines. The quercetin has many health benefits including protection against various viral diseases, cardiovascular diseases, osteoporosis, and multiple cancers. This pleiotropic compound has functional groups which interact with different cellular targets and interrupt various cellar pathways. Earlier studies which have established the antiviral activities of quercetin shall pave way to develop effective, safe, and affordable natural formulations for the treatment and prophylaxis of novel coronavirus. This review has mainly focused on the perspective of developing quercetin-based SARS-CoV2 drugs, based on its ability to inhibit the key enzymes of the virus. The proven role of zinc in the suppression of RdRp, leading to the inhibition of viral replication, suggests its therapeutic value against SARS-CoV-2 infection. Mechanisms underlying the expression of candidate miRNA genes in the infection process, and the roles of quercetin in their regulation need to be assessed using *in vitro* and *in vivo* systems. A couple of clinical trials on using quercetin for COVID-19 treatment are in pipeline, yet extensive works have to be urgently taken up to formulate quercetin-based drugs. Further, this paper provides proof of concept by showing the docking of key proteins from SARS-CoV-2 with quercetin. All the key proteins experimented through molecular docking have docked at multiple residues with satisfactory binding energies, showing that quercetin is a potential drug molecule; further research has to be urgently diverted to *in vitro* and *in vivo* model systems to enable the use of quercetin-based drugs at the earliest.

## Data Availability

There are no additional data associated with this study.

## Abbreviations

COVID-19: Coronavirus disease 2019
SARS-CoV-2: Severe acute respiratory syndrome coronavirus 2
CASTp: Computed Atlas of Surface Topography of proteins
3CL^pro^: 3C-like protease
RdRp: RNA-dependent RNA polymerase
